# Nurses for Public Health Work

**Published:** 1924-11

**Authors:** 


### NURSES FOR PUBLIC HEALTH WORK.

Since 1920 an international course of training in Public Health Nursing, organised by the Red Cross Societies, has been held at Bedford College for Women in order to meet the increasing demand in all countries for nurses qualified for service in the Public Health field. This year the students have more than maintained the high standard set by former groups, and all sixteen students received the special League Certificate, having done most excellent work throughout the year; in addition, nine of the students, owing to their good knowledge of the English language, were allowed to enter for the Board of Education Examination (Bedford College Certificate), and all passed successfully. The course, as arranged, makes it possible for a well educated and capable woman who has organising ability and is really interested in the development of nursing, but who lacks the technical training which she would certainly have sought had nursing facilities existed in her country, to return from London sufficiently well equipped to constitute a staunch advocate and a potential organiser of nurses' training schools in her own land.

### Two Courses.

Nor must the international value of this work be lost sight of—it is perhaps one of the most practical efforts being made to promote the ideals of the League of Nations. For nine months of the year these women, brought from all parts of the world, live under one roof and attend the same college course on Health and Social Science; problems common to all humanity and not to one nation in particular are studied and discussed, and racial differences and politics sink entirely into the background, although at the same time the value of each national point of view can be fully appreciated. This year there are to be two distinct courses for the Internationals, one for Public Health Nursing and one for Hospital Administration, the latter having been arranged with a view to giving senior nurses holding executive and teaching positions in their own countries an opportunity for studying the subject of Training School and Hospital direction in all its aspects. It is hoped that one result of these two sister courses being carried on side by side will be to demonstrate that there is in fact no hard-and-fast line dividing the work of the public health nurse from that of the nurse working in hospital, and that they are both in reality public health workers.

---

**British Medical Association's Handbook.**

**The British Medical Association has issued its handbook for 1924–25 (2s. 6d.). The handbook is a reference volume for honorary secretaries and other workers of the Association and all who are interested in its constitution, work and history.**

